# Outbreak of SARS-CoV-2 in a geriatric acute-care ward during summer 2023: current aspects of infection control in the postpandemic period

**DOI:** 10.3205/dgkh000457

**Published:** 2024-01-30

**Authors:** Bettina Lange, Gracinda Mesquita, Heinrich Burkhardt, Marlis Gerigk, Alexandra Heininger

**Affiliations:** 1 Medical Faculty Mannheim, Heidelberg University, University Medical Center Mannheim, Department of Hygiene, Mannheim, Germany; 2 Medical Faculty Mannheim, Heidelberg University, University Medical Center Mannheim, 4th Department of Medicine, Mannheim, Germany; 3 Marlis Gerigk Medical Faculty Mannheim, Heidelberg University, University Medical Center Mannheim, Institute für Medical Microbiology and Hygiene, Mannheim, Germany

**Keywords:** SARS-CoV-2, outbreak, geriatrics, post-pandemic, management

## Abstract

**Aim:**

Management of a SARS-CoV-2 outbreak in geriatric patients, taking into account the transition to the post-pandemic period.

**Methods:**

PCR tests were conducted to identify the scale of infection during the outbreak; no new patients were admitted to the ward until the availability of the PCR results. Based on the results and individual risk assessment, three cohorts were formed and treated as recommended by the RKI. After terminating the admissions stop, new admissions received PCR screening. Contact patients were retested on days 3 and 5. Employees carried out self-monitoring, and if symptoms developed, an antigen test was performed.

**Results:**

Nine of the 11 PCR-positive patients (6m, 5f), median age 85 years, were immunized. Eight patients were symptomatic, ten received antiviral therapy and two required intensive care. Three symptomatic employees had a positive antigen test. Patients without direct contact to the positive cases who initially tested negative and the 16 new admissions with a negative PCR test did not contract COVID-19. Outbreak management ended after 15 days without deaths from COVID-19.

**Conclusion:**

During the outbreak, PCR screening, the temporary stop in new admission until the availability of PCR results, and the risk-adapted cohorting of patients supplemented by consistent PCR tests of new admissions formed the basis for successful outbreak management. Treatment can be made possible despite high vulnerability. Close symptom monitoring and rapid implementation of measures reduce the risk. Repeated PCRs of direct-contact patients on day 3 can warrant pre-emptive antiviral therapy despite being asymptomatic; testing on day 5 makes it possible to shorten preventive isolation measures. The use of protective masks and self-monitoring by employees are fundamental to preventing further infections.

## Zusammenfassung

**Hintergrund:**

Management eines SARS-CoV-2-Ausbruchs bei geriatrischen Patienten unter Berücksichtigung des Übergangs in die endemische Situation.

**Methoden:**

Im Ausbruch erfolgte zur Klärung der Infektionssituation ein PCR-Screening und bis zum Vorliegen der Ergebnisse ein kurzer Aufnahme-Stopp. Anhand der PCR-Ergebnisse wurden drei risikoadaptierte Kohorten gebildet, die gemäß RKI behandelt wurden. Mit Ende des Aufnahme-Stopps erhielten Neuaufnahmen ein PCR-Screening. Kontaktpatienten wurden an Tag 3 und 5 erneut getestet. Mitarbeiter führten ein Selbstmonitoring und bei Symptomen einen Antigentest durch.

**Ergebnisse:**

Neun der 11 PCR-positiven Patienten (6m, 5w), Altersmedian 85 Jahre, waren grundimmunisiert. Symptomatisch waren acht, eine antivirale Therapie erhielten 10 Patienten. Zwei wurden intensivmedizinisch behandelt. Drei symptomatische Mitarbeiter hatten einen positiven Antigentest. Alle initial PCR-negativ getesteten Patienten ohne direkten Kontakt und 16 PCR-negativ getestete Neuaufnahmen erkrankten nicht an COVID-19. Das Ausbruchmanagement endete nach 15 Tagen. Kein Patient verstarb an COVID-19.

**Diskussion:**

Im Ausbruch sind PCR-Screening, ein passagerer Aufnahme-Stopp bis zum Vorliegen der Ergebnisse und die risikoadaptierte Kohortierung der Patienten die Basis für ein erfolgreiches Ausbruchmanagement. Ergänzt durch ein konsequentes Aufnahmescreening kann die Behandlung trotz hoher Vulnerabilität ermöglicht werden. Engmaschige Symptombeobachtung und zeitnahes Handeln senken das Risiko. Die PCR-Testung direkter Kontaktpatienten an Tag 3 dient bei positivem Resultat der Veranlassung einer präemptiven antiviralen Therapie begründen; Testung an Tag 5 dient der Verkürzung der Quarantäne. Mitarbeiter sollten Schutzmasken tragen, um – ergänzt durch ein Selbstmonitoring – weitere Ansteckungen zu verhindern.

## Introduction

On 7 April 2023, the last legal Corona protection measures for hospitals and nursing homes in Germany were suspended according to § 28b of the German Infection Protection Act. This marked the beginning of prevention adaptation from a pandemic to an endemic situation [1]. Almost simultaneously, on 5 May 2023, the World Health Organization (WHO) declared the end of the COVID-19 pandemic, which cost nearly 7 million human lives since 2020 [2]. Later in the year, acute respiratory illness (ARE) activity was at low levels, which was usually seen in the summer of the pre-pandemic period [3]. The rate of basic immunization on 8 August was 76.4% in the total population and as high as 90.1% in the subgroup of people over 60 years of age in Germany [4].

From calendar week (CW) 28, an increase in reports of laboratory-confirmed COVID-19 cases was registered again for the first time by the Robert Koch Institute (RKI). This was associated with the new omicron variant EG.5, first designated as a variant of interest (VOI) under observation by WHO on 9 August [5]. While an ongoing increase of the prevalence of EG.5 was reported in September 2023 [6], no change in disease severity was observed compared with other omicron variants [7]. The proportion of patients hospitalized for COVID-19 in week 33 was 9% [8]. At this time, the 7-day incidence was highest in the age group 80 years and older, at 14 per 100,000 individuals [8].

At that time, a paper on the integration of SARS-CoV-2 as a causative agent of infections in the endemic situation into the previously valid recommendations for “Infection prevention in the context of care and treatment of patients with communicable diseases” from 2015 [9] by the German Commission for Hospital Hygiene and Infection Prevention (KRINKO) at the Robert Koch Institute was already in the publication process. 

We report our management of a SARS-CoV-2 outbreak within a vulnerable cohort of geriatric patients considering transition to endemicity.

## Methods

In week 33, four of 19 patients in our acute geriatric ward exhibited typical symptoms of COVID-19 disease, which was confirmed by PCR (cases 1, 2, 3, and 4). The room neighbor of case 3 was still questionably positive at this time with a Ct value of 39 (case 4). In accordance with Section 6(3) of the German Infection Protection Act, an outbreak was established and the city health authority was informed.

In view of the outbreak situation, the remaining 15 patients were screened immediately using PCR tests to clarify their infection status, although they had no symptoms. No further patients were admitted to the ward until the results were available on the following day. On the next day, the event-related PCR screening showed that a total of 10 SARS-CoV-2-positive and 9 SARS-CoV-2-negative patients were currently present in this ward.

The PCR-based overview of infection status formed the basis for classifying all patients in the outbreak ward into three cohorts according to KRINKO recommendations for the management of infection outbreaks [10]. Cohort I consisted of PCR-positive patients; cohort II PCR-negative patients who had direct unprotected contact with positively tested fellow patients as room neighbors; and cohort III comprised PCR-negative patients without direct contact with infected fellow patients.

According to RKI [11], the following procedure was applied in the cohorts:

- Cohort I: Isolation in a single room or in a cohort; termination of isolation after seven days without further testing if the patient was symptom-free.
- Cohort II: Quarantine for five days with symptom monitoring and control PCR on day 3, in order to be able to initiate preemptive antiviral therapy [12] at an early stage if the test result was positive. On day 5, another PCR was performed as a prerequisite for lifting the quarantine..
- Cohort III: Symptom monitoring.

Table 1 (Tab. 1) presents the other precautions that were implemented as basic measures throughout the period and the procedure depending on the infection status of the patients. Additional measures to maintain separation between the three cohorts consisted of spatial separation in two areas of the ward and supervision by assigned nursing staff (Table 1 (Tab. 1)).

After this delineation of cohorts, the admission freeze was terminated within 24 hours. However, all new patients were tested for SARS-CoV-2 by point-of-care testing (POCT-PCR) before admission to a patient room. Positively tested new patients were assigned to cohort I on a nursing basis. Negatively tested, asymptomatic patients were placed in a freshly processed room and formed cohort IV; they were cared for together with cohort III.

Asymptomatic employees were not screened; rather, they independently monitored themselves for possible COVID-19 symptoms. In case of symptoms, an antigen test was performed, and work was promptly stopped. Employees were allowed to resume work after five days at the earliest if they were symptom-free and the antigen test was negative [13].

## Results

In the SARS-CoV-2 outbreak, 10 geriatric patients (five men, five women) initially tested positive. One male patient out of initially three PCR-negative direct-contact patients (cohort II) tested positive for SARS-CoV-2 using PCR on day three (case 11). The median age of the 11 positive cases was 85 years (range, 69–91); five of these were hospitalized for early rehabilitation complex treatment. Known risk factors for a severe course were hypertension (n=9), diabetes mellitus (n=3) and chronic obstructive pulmonary disease (COPD) (n=1); a cognitive deficit was additionally found in six patients. Complete vaccination according to the recommendation of the German Standing Commission on Vaccination (STIKO) at the Robert Koch Institute was ascertainable for nine of the 11 cases (82%); three patients had had additional booster vaccinations [14]; one patient had an incomplete vaccination and another one could not provide his vaccination information (Table 2 (Tab. 2)).

Eight of the 11 patients who tested positive developed COVID-19 symptoms (fever, cough); 10 received antiviral therapy (nirmatrelvir + ritonavir). One patient (case 8) with bacterial superinfected pneumonia required intubation and ventilation in an intensive care unit for five days; another (case 10) patient suffering from chronic lung disease needed supportive oxygen therapy in the intermediate care unit for one night (Table 2 (Tab. 2)).

The asymptomatic contact patient who tested positive on day three received preemptive antiviral therapy on the same day and could be discharged to home care. The other two contact patients remained PCR negative: one patient was discharged promptly to home care, and the other patient required further hospitalization due to a complicated urinary tract infection. The two PCR-positive, symptomatic new admissions showed rapid improvement under early antiviral therapy.

Three staff members with direct contact with PCR-positive patients in the outbreak cohort showed typical COVID-19 symptoms and a positive antigen test during the first week of the outbreak, despite basic immunization.

In the group patients whose initial PCR test was negative (cohort III) as well as in the 16 new admissions whose PCR test was negative (cohort IV), no COVID-19 disease occurred during an observation period of two weeks. Accordingly, outbreak management measures could be terminated after 15 days. No patient died from COVID-19 during the outbreak described above (Table 2 (Tab. 2)).

## Discussion

Successful management of a SARS-CoV-2 outbreak is based on rapid recognition of the triggering event, systematic initiation of acute measures, and immediate root-cause analysis [10]. The vulnerable collective of geriatric patients with advanced age and numerous concomitant diseases also poses a challenge for both hospitals and care facilities in the endemic situation. With the declaration of a SARS-CoV-2 outbreak, immediate event-based PCR screening of all patients present in the outbreak ward provides the basis for risk-adapted cohorting of the patients involved into three groups. These prerequisites allow additional patients to receive treatment under safe conditions after a short-term admissions halt, despite high vulnerability. Correspondingly, during the outbreak, newly admitted patients should receive an admission screening for SARS-CoV-2 to enable cohort-based care. With these measures, no patient in our own cohort who initially tested PCR-negative developed COVID-19.

According to KRINKO recommendations, patients with aerogenic infection should be promptly isolated or clustered [9]; the indication for antiviral therapy should always be reviewed early in the interest of the individual patient. In our own collective, two male patients of the outbreak cohort (cases 8 and 10, 83 and 72 years of age, respectively) showed a severe course and needed intensive medical treatment, despite complete vaccination according to STIKO and antiviral therapy (case 8), and known risk factors (hypertension in both cases 8 and 10), diabetes mellitus (case 8), chronic respiratory insufficiency (case 10). Even in the endemic situation with the currently dominant omicron variant EG.5, severe courses must be expected at any time in a vulnerable cohort of geriatric patients who possess numerous risk factors. Therefore, close symptom monitoring is highly important to enable implementing all reasonable therapeutic options up to intensive medical therapy at the earliest. As a consequence, we decided to perform additional PCR tests on direct contacts with a high risk of a severe course on days 3 and 5 to make the initiation of preemptive therapy possible at an early stage, in the event of a positive result. In case of signs of infection of unclear origin, a SARS-CoV-2 PCR test should be repeated even in initially PCR-negative contact patients before lifting the quarantine to exclude infection.

Employees should immediately begin wearing FFP2 masks or surgical masks as soon as a SARS-CoV-2 outbreak is suspected [13]. Well-fitting FFP2 masks were also preferred in our own collective from the point of view of self-protection. For direct contact with COVID-19 patients, personal protective equipment is applied (extended) on a pathogen-specific basis [11]. Self-monitoring of staff for possible COVID-19 symptoms was sufficient to prevent infection within the staff group or additional patients.

In the course of the root-cause analysis, the following clues about the source of the outbreak emerged: a patient with mild symptoms was not immediately tested using PCR diagnostics, and one visitor subsequently revealed that she had experienced mild symptoms but had not used a protective mask. Furthermore, a staff member with direct patient contact developed symptoms and tested positive in the antigen test two days after the onset of the outbreak; thus an infection in the asymptomatic stage could also have originated from her. Consistent, rapid PCR testing of symptomatic patients, strict limitation of visits by symptomatic relatives, and antigen testing of symptomatic staff contributed significantly to the rapid limitation of the outbreak in this reported outbreak.

The validity of our data is limited by the retrospective nature and the current lack of results of mutational analysis of the SARS-CoV-2 strain in our patient population. To our knowledge, this is the first report of successful outbreak management in in-house patients of a geriatric center in the postpandemic phase.

## Conclusions

Consistent monitoring of typical COVID-19 symptoms in patients and staff, rapid PCR testing of all patients present in the outbreak ward, symptom-based antigen testing of staff, consistent wearing of protective masks, and early review of the indication for antiviral therapy are the critical tools, even in the post-pandemic phase, for timely and successful termination of a SARS-CoV-2 outbreak in a vulnerable cohort of geriatric patients.

## Notes

### Competing interests

The authors declare that they have no competing interests.

### Funding

B. Lange, G. Mesquita, H. Burkhardt, M. Gerigk and A. Heininger affirm that there are no financial or non-financial interests that are directly or indirectly related to the work submitted for publication.

### Compliance with ethical standards

The authors confirm that the study was approved by the Research Ethics Committee of the Medical Faculty Mannheim, Heidelberg University, Germany (No. EK-II-02/2023), and certify that the study was performed in accordance with the ethical standards as laid down in the 1964 Declaration of Helsinki and its later amendments.

## Figures and Tables

**Table 1:**
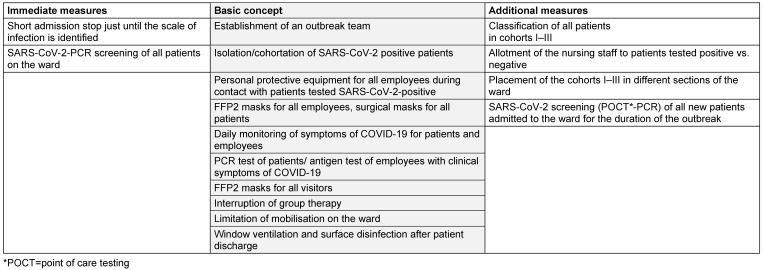
Management of a SARS-CoV-2 outbreak in a geriatric acute care ward, August 2023

**Table 2:**
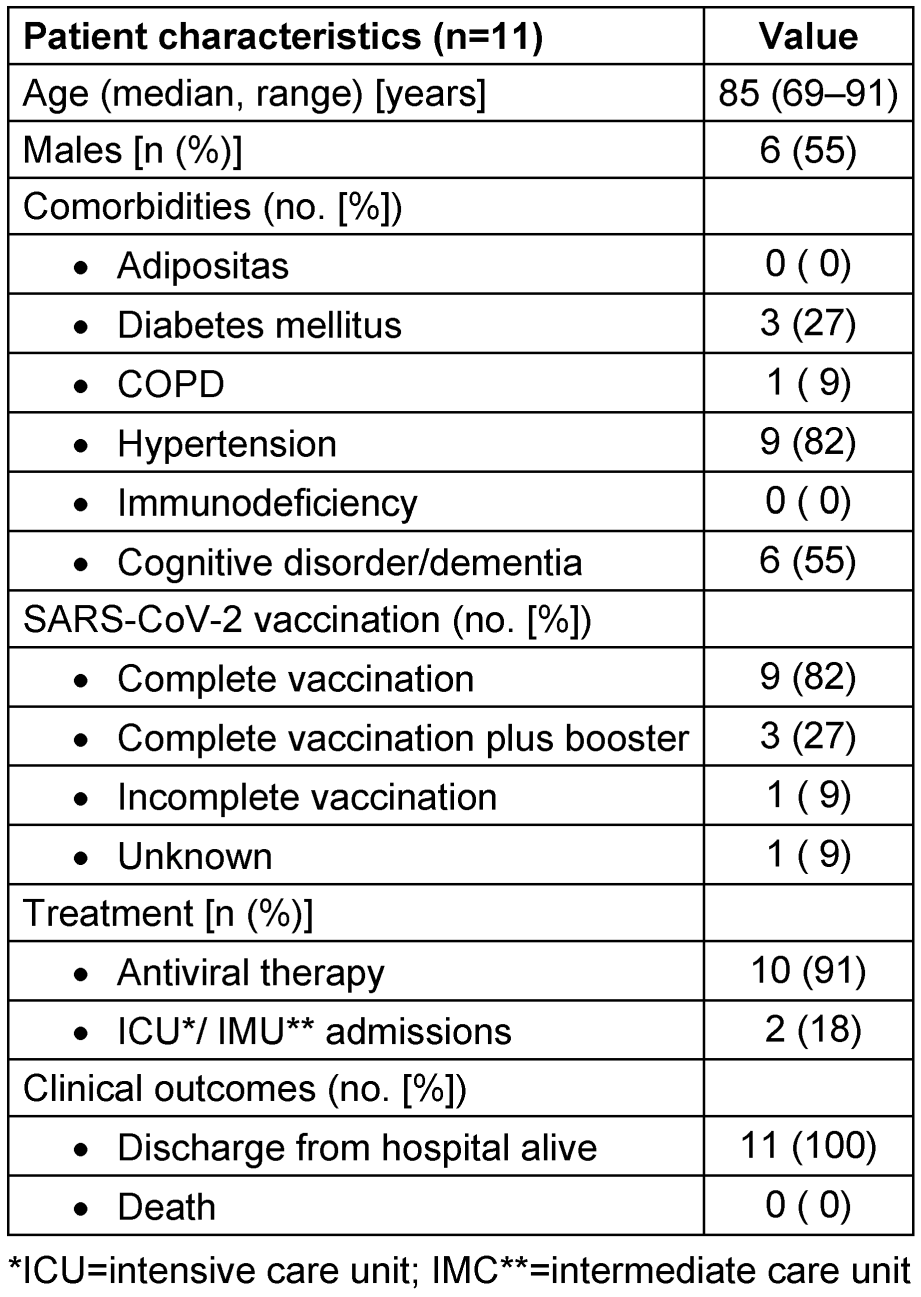
Demographic and clinical characteristics, and outcome of geriatric patients during an outbreak, August 2023
